# Female Sex Is Associated with Worse Prognosis in Patients with Hypertrophic Cardiomyopathy in China

**DOI:** 10.1371/journal.pone.0102969

**Published:** 2014-07-21

**Authors:** Yilu Wang, Jizheng Wang, Yubao Zou, Jingru Bao, Kai Sun, Ling Zhu, Tao Tian, Hu Shen, Xianliang Zhou, Ferhaan Ahmad, Rutai Hui, Lei Song

**Affiliations:** 1 Department of ICU, China Meitan General Hospital, Beijing, China; 2 Sino-German Laboratory for Molecular Medicine, State Key Laboratory of Cardiovascular Disease, Fuwai Hospital, National Center for Cardiovascular Disease, Chinese Academy of Medical Sciences and Peking Union Medical College, Beijing, China; 3 Department of Cardiovascular Internal Medicine, State Key Laboratory of Cardiovascular Disease, Fuwai Hospital, National Center for Cardiovascular Disease, Chinese Academy of Medical Sciences and Peking Union Medical College, Beijing, China; 4 Center for cardiovascular diseases, PLA Navy General Hospital, Beijing, China; 5 Department of Cardiovascular Medicine, First Affiliated Hospital of Medical College, Xi’an Jiaotong University, Xi’an, Shanxi Province, China; 6 Hypertension Center, State Key Laboratory of Cardiovascular Disease, Fuwai Hospital, National Center for Cardiovascular Disease, Chinese Academy of Medical Sciences and Peking Union Medical College, Beijing, China; 7 Division of Cardiovascular Medicine, Department of Internal Medicine, University of Iowa Carver College of Medicine, Iowa City, Iowa, United States of America; Washington Hospital Center, United States of America

## Abstract

**Background:**

Sex plays an important role in the clinical expression and prognosis of various cardiovascular diseases. This study was designed to observe the effects of sex on hypertrophic cardiomyopathy (HCM).

**Methods and Results:**

A total of 621 unrelated patients with HCM without heart failure (460 males) were enrolled from 1999 to 2011. Compared to male patients, at baseline female patients were older at diagnosis (49.6±17.2 years vs. 46.7±14.4 years, *P = *0.033), and had greater frequency of left ventricular outflow tract obstruction (72/161, 44.7% vs. 149/460, 32.4%, *P = *0.005). During the average four year follow-up period (range 2–7 years), survival analysis showed that the incidences of mortality from all causes, cardiovascular death and progression to chronic heart failure were greater in women than in men (*P = *0.031, 0.040 and 0.012, respectively). After adjustment for multiple factors that may confound survival and cardiac function, female sex remained an independent risk factor for all-cause mortality, cardiovascular death, and chronic heart failure [hazard ratio (HR) 2.19, 95% confidence interval (CI) 1.21–3.95, *P = *0.010; HR 2.19, 95% CI 1.17–4.09, *P = *0.014; HR 1.73, 95% CI 1.12–2.69, *P = *0.014, respectively] in HCM patients. Subgroup analysis revealed that female sex as a risk factor was identified only in patients younger than 50 years old (*P = *0.011, 0.011 and 0.009, respectively), but not for those 50 years or older.

**Conclusion:**

Our results suggest that female sex is associated with worse survival and heart failure in HCM patients. Further studies are required to determine whether female hormones modify the clinical expression and prognosis of HCM.

## Introduction

Hypertrophic cardiomyopathy (HCM) affects 1 in 500 people, making it the most common heritable heart disease, and is strongly associated with sudden cardiac death (SCD) in young adults. [1] The clinical expression and natural history of HCM are extremely heterogeneous, with patients being either asymptomatic or having multiple symptoms, displaying left ventricular wall thickness ranging from normal to extremely hypertrophic, having normal left ventricular outflow tract (LVOT) gradients to those with severe obstruction, and a normal lifespan to refractory heart failure or SCD as the first manifestation. [2]–[4] Sex is known to impact many aspects of cardiovascular diseases, including prevalence, severity of clinical manifestations, and outcomes. [5]–[8] Several studies have yielded divergent associations of female sex with improved, unchanged, or worsened prognosis. [7]–[10] This study was designed to investigate the effect of sex on clinical expression and prognosis in Chinese HCM patients.

## Methods

### Patients

Consecutive unrelated patients with HCM were diagnosed at Fuwai Hospital, Chinese Academy of Medical Sciences during the period of 1999 to 2011. All patients underwent a complete cardiac evaluation, including a detailed history and clinical examination, 12-lead electrocardiogram, echocardiogram and/or cardiac magnetic resonance imaging. HCM was ascertained as maximum left ventricular wall thickness ≥15 mm (or ≥13 mm with a family history of HCM) in the absence of any other evident cardiac or systemic disease capable of producing similar hypertrophy magnitudes, such as uncontrolled hypertension (home blood pressure monitoring ≥140/90 mmHg), cardiac valve disease, congenital heart disease or amyloidosis. [2], [3] Patients with severe chronic heart failure (New York Heart Association functional class III or IV) at enrollment were excluded. This study was performed in accordance with the principle of the Declaration of Helsinki and approved by the Ethics Committees of Fuwai Hospital. Written informed consent was provided by all participants.

### Clinical Outcomes

The primary outcomes were mortality from all causes, comprised of non-cardiovascular death and cardiovascular death, including SCD, heart failure-related death and fatal stroke. SCD was defined as sudden and unexpected death within one hour from symptom onset in patients who previously experienced a relatively stable or uneventful clinical course. Progression to chronic heart failure (NYHA III or IV), ventricular tachycardia and/or fibrillation (VT/VF), implantable cardioverter defibrillator (ICD) discharge, non-fatal stroke, atrial fibrillation (AF), myocardial infarction, transient heart failure, implantation of ICD and septal reduction therapy (including myectomy and alcohol ablation) during follow-up were recorded as secondary events. Chronic heart failure was diagnosed on the basis of shortness of breath at rest or during exertion, and/or fatigue; signs of fluid retention such as ankle swelling; and objective evidence of an abnormality in the heart structure or function at rest. [11] The severity of heart failure was assessed according to the NYHA functional classification. Transient heart failure referred to symptomatic heart failure over a limited time period during follow-up. [11] The follow-up period ended at the time of death or in January 2012 (the last time of follow-up).

### Statistical Analysis

Normally distributed and skewed distributed data were expressed as mean ± SD (standard deviation) and median (25th–75th percentile), and were tested using unpaired Student’s *t*-test and non-parameter test, respectively. The chi-square test was utilized to compare non-continuous variables that were expressed as proportions. Survival curves were constructed according to the Kaplan-Meier method, and comparisons were performed using the log-rank test. Both univariate and multivariate Cox proportional hazard regression models were used to calculate relative risks and 95% confidence intervals. The factors chosen for adjustment were age, syncope (without any invasive treatment, including implantation of ICD, pacemaker and septal reduction therapy), SCD family history, maximal left ventricular wall thickness, left atrial diameter, AF, left ventricular outflow tract (LVOT) obstruction (without septal reduction therapy) and NYHA functional class (II compared to I) at enrollment. [12], [13] Multivariate analyses were performed with a stepwise forward regression model, in which each variable with a *P* value ≤0.05 based on univariate analysis was entered into the model. *P* <0.05 was considered as statistically significant. Statistical analysis was performed with SPSS software, version 13.0 (SPSS Inc. Chicago, USA).

## Results

### Clinical Characteristics of Patients

A total of 621 HCM patients were selected, including 460 males and 161 females (male female ratio 2.9∶1). Female patients were older at the time of diagnosis than male patients (49.6±17.2 years vs. 46.7±14.4 years, *P = *0.033), and had a lower frequency of abnormal T waves on electrocardiogram (98/161, 60.9% vs. 336/460, 73.0%, *P = *0.004). The left ventricular end-diastolic diameter in female patients was significantly smaller than in male patients (42.3±5.8 mm vs. 45.4±5.8 mm, *P* < 0.001), although the left atrial diameters were similar between the two groups. LVOT obstruction (gradient ≥30 mmHg at rest) was more frequently identified in female patients than in male patients (72/161, 44.7% vs. 149/460, 32.4%, *P = *0.005) (Table 1). The pressure gradient was significantly higher in females than in males among patients with LVOT obstruction (81.9±41.0 mmHg vs. 71.9±27.3 mmHg, *P = *0.034).

**Table 1:** Baseline Clinical Features of 621 HCM Patients According to Sex*.

Parameters	Overall	Male Patients	Female Patients	*P* Value
**Number of patients**	621	460(74.1%)	161 (25.9%)	
**Age at diagnosis (yrs)**	47.5±15.2	46.7±14.4	49.6±17.2	***0.033***
**Syncope**	149 (24.0%)	110 (23.9%)	39 (24.2%)	0.937
**Coronary artery disease**	88 (14.2%)	67 (14.6%)	21 (13.0%)	0.634
**HCM family history**	140 (22.5%)	99 (21.5%)	41 (25.5%)	0.303
**SCD family history**	81 (13.0%)	56 (12.2%)	25 (15.5%)	0.277
**Heart rate (bpm)**	70.2±11.3	70.6±11.6	69.1±10.2	0.13
**Systolic blood pressure (mmHg)**	122.0±17.2	122.6±16.5	120.4±19.1	0.189
**Diastolic blood pressure (mmHg)**	75.0±10.4	76.1±10.5	71.9±9.6	***<0.001***
**Pathological Q wave**	139 (22.4%)	96 (20.9%)	43 (26.7%)	0.126
**T wave inversion/Giant T wave**	434 (69.9%)	336 (73.0%)	98 (60.9%)	***0.004***
**Maximum LV wall thickness (mm)**	21.0±4.7	21.0±4.8	21.0±4.1	0.728
**Left atrial diameter (mm)**	39.8±6.7	40.0±6.8	39.2±6.3	0.207
**LVEDD (mm)**	44.6±5.9	45.4±5.8	42.3±5.8	***<0.001***
**EF (%)**	67.4±8.0	67.5±7.8	67.1±8.5	0.61
**LVOT gradient ≥30 mmHg**	221 (35.6%)	149 (32.4%)	72 (44.7%)	***0.005***
**NYHA II**	267 (43.0%)	188 (40.9%)	79 (49.1%)	0.071
**VT/VF**	7 (1.1%)	4 (0.9%)	3 (1.9%)	0.304
**Atrial fibrillation**	78 (12.6%)	59 (12.8%)	19 (11.8%)	0.736
**Stroke**	12 (1.9%)	10 (2.2%)	2 (1.2%)	0.46
**Transient HF**	13 (2.1%)	9 (2.0%)	4 (2.5%)	0.687
**Acute myocardial infarction**	3 (0.5%)	2 (0.4%)	1 (0.6%)	0.769
**Surgical septal myectomy**	11 (1.8%)	5 (1.1%)	6 (3.7%)	0.029
**Alcohol septal ablation**	77 (12.4%)	55 (12.0%)	22 (13.7%)	0.571
**ICD implantation**	4 (0.6%)	3 (0.7%)	1 (0.6%)	0.966
**Appropriate ICD discharge**	4 (0.6%)	3 (0.7%)	1 (0.6%)	0.966
**Pacemaker implantation**	49 (7.9%)	31 (6.7%)	18 (11.2%)	0.072
**Medication**				
** β-blocker**	424(68.3%)	316(68.7%)	108(67.1%)	0.705
** Calcium channel blocker**	232(37.4%)	170(37.0%)	62(38.5%)	0.726

HCM = hypertrophic cardiomyopathy, SCD = sudden cardiac death, Max LV = maximum left ventricular, LVEDD = left ventricular end-diastolic diameter, LVMI = left ventricular mass index, EF = ejection fraction, LVOT = left ventricular outflow tract, NYHA = New York Heart Association, VT/VF = ventricular tachychadia/fibrillation, HF = heart failure, ICD = implantable cardioverter defibrillator.

*Data with normal distribution are presented as mean±SD (standard deviation), while data with skewed distribution were presented as median (25–75% percentile), non-continuous variables expressed as proportions.

### Clinical Outcomes in Female and Male HCM Patients

During the average four year (range 2–7 years) follow-up, 47 (7.6% of total) patients died, with 89% (42/47) of deaths due to cardiovascular-related causes, including 19 SCDs, 15 heart failure-related death and 8 fatal strokes (Table 2). Five patients died of non-cardiovascular causes, including lung cancer (n = 2), liver cancer (n = 1), rectal cancer (n = 1) and renal failure (n = 1). The annual incidence of cardiovascular death was 1.34% in the entire HCM cohort. The all cause and cardiovascular mortality rates were both higher in females than in males (19/161, 11.8% vs. 28/460, 6.1%, *P = *0.018 and 17/161, 10.6% vs. 25/460, 7.6%, *P = *0.026, respectively) (Table 2). Female patients developed heart failure more frequently than male patients (34/161, 21.1% vs. 55/460, 12.0%, *P = *0.004).

**Table 2:** Cardiovascular Events and Invasive Treatments During Follow-up According to Sex*.

Events	Overall	Male Patients	Female Patients	*P* Value
**All-cause death**	47/621 (7.6%)	28/460 (6.1%)	19/161 (11.8%)	***0.018***
**Cardiovascular death**	42/621 (6.8%)	25/460 (5.4%)	17/161 (10.6%)	***0.026***
**Sudden death**	19/621 (3.1%)	12/460 (2.6%)	7/161 (4.3%)	0.27
**HF-related death**	15/621 (2.4%)	9/460 (2.0%)	6/161 (3.7%)	0.208
**Fatal stroke**	8/621 (1.3%)	4/460 (0.9%)	4/161 (2.5%)	0.118
**NYHA III/IV**	89/621 (14.3%)	55/460 (12.0%)	34/161 (21.1%)	***0.004***
**VT/VF**	4/614 (0.7%)	3/456 (0.7%)	1/158 (0.6%)	0.973
**Appropriate ICD discharge**	1/617 (0.2%)	1/457 (0.2%)	0/160 (0.0%)	0.554
**Atrial fibrillation**	40/543 (7.4%)	28/401 (7.0%)	12/142 (8.5%)	0.565
**Stroke**	29/609 (4.8%)	19/450 (4.2%)	10/159 (6.3%)	0.293
**Transient HF**	30/608 (4.9%)	20/451 (4.4%)	10/157 (6.4%)	0.335
**Acute myocardial infarction**	1/618 (0.2%)	1/458 (0.2%)	0/160 (0.0%)	0.554
**Surgical septal myectomy**	13/610 (2.1%)	12/455 (2.6%)	1/155 (0.6%)	0.138
**Alcohol septal ablation**	25/544 (4.6%)	22/405 (5.4%)	3/139 (2.2%)	0.112
**ICD implantation**	5/617 (0.8%)	4/457 (0.9%)	1/160 (0.6%)	0.761
**Pacemaker implantation**	34/572 (5.9%)	26/429 (6.1%)	8/143 (5.6%)	0.838

HF = heart failure, VT/VF = ventricular tachychadia/fibrillation, ICD = implantable cardioverter defibrillator, NYHA = New York Heart Association.

*Proportion of patients with the event happened or received the treatment during follow-up (excluding patients with the specific event or received the specific treatment at initial evaluation).

**Figure 1:**
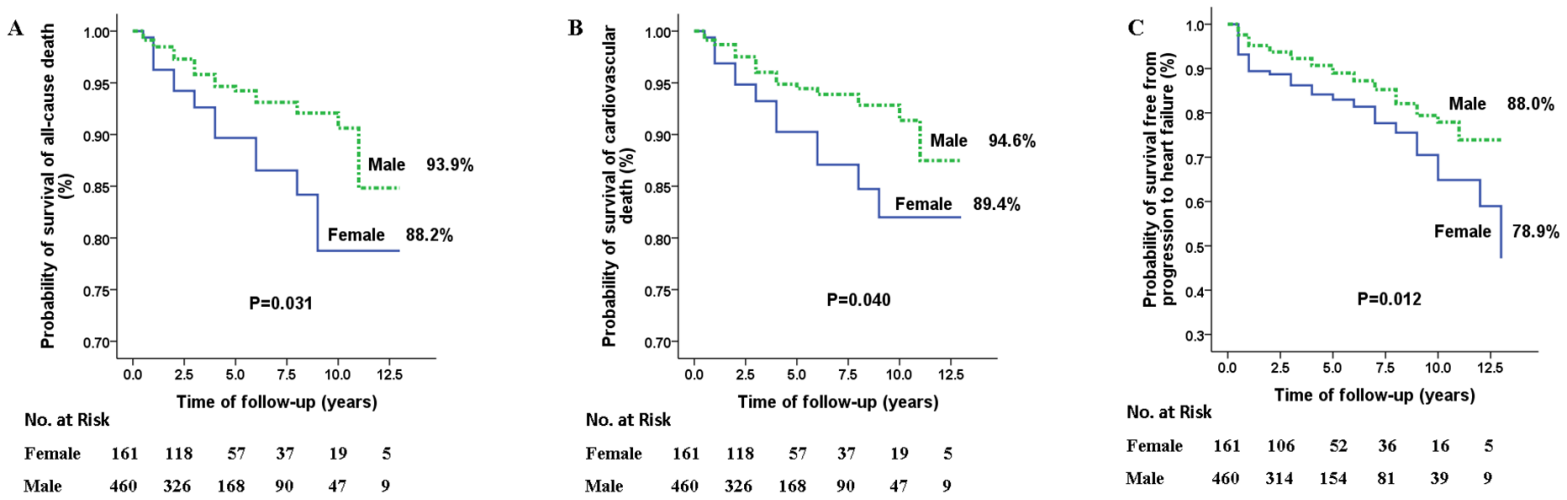
Survival in males and females free from death from all causes (A), cardiovascular death (B) and progression to heart failure (C). Female patients exhibited higher rates of death from all causes, cardiovascular death and progression to heart failure than male patients. The P values were calculated by log-rank test.

Kaplan-Meier analysis showed that female patients had a significantly higher risk of all-cause death, cardiovascular death, and progression to heart failure (*P = *0.031, 0.040 and 0.012, respectively) relative to male patients (Figure 1). Univariate Cox proportional hazard regression models showed that females had 87% higher risk of all-cause mortality (HR 1.87, 95% CI 1.04–3.35, *P = *0.035), 88% higher risk of cardiovascular death (HR 1.88, 95% CI 1.02–3.48, *P = *0.045), and 71% higher risk of progression to heart failure (HR 1.71, 95% CI 1.12–2.63, *P = *0.014) than did males. The increased risk in women remained significant in multiple Cox proportional hazard regression models, adjusted for baseline clinical variables including age, syncope (without any invasive treatment, including implantation of ICD, pacemaker and septal reduction therapy), SCD family history, maximum left ventricular wall thickness, left atrial diameter, AF, LVOT obstruction (without septal reduction therapy) and NYHA functional class (II compared to I) at enrollment (all-cause death HR 2.19, 95% CI 1.21–3.95, p = 0.010; cardiovascular death HR 2.19, 95% CI 1.17–4.09, p = 0.014; progression to heart failure HR 1.73, 95%CI 1.12–2.69, p = 0.014, respectively) (Table 3).

**Table 3:** Relation Between Clinical Variables at Initial Evaluation and Outcomes*.

	Overall death		Cardiovascular death		SCD		Chronic HF		Stroke	
	Relative risk	*P* Value	Relative risk	*P* Value	Relative risk	*P* Value	Relative risk	*P* Value	Relative risk	*P* Value
Variables	(95% CI)		(95% CI)		(95% CI)		(95% CI)		(95% CI)	
**Female sex**	2.19	***0.01***	2.19	***0.014***	–	0.3	1.73	***0.014***	–	0.458
	1.21–3.95		1.17–4.09				1.12–2.69			
**Age at enrollment**	–	0.144	–	0.415	–	0.378	–	0.195	1.05	***0.001***
									1.02–1.08	
**Syncope** &	–	0.158	–	0.233	–	0.753	–	0.537	–	0.535
**SCD family history**	–	0.215	–	0.116	3.32	***0.015***	–	0.272	–	0.643
					1.26–8.73					
**Maximum LV wall thickness**	–	0.5	–	0.832	–	0.449	–	0.786	–	0.729
**Left atrial diameter**	1.09	***<0.001***	1.09	***<0.001***	–	0.11	1.04	***0.022***	1.07	***0.008***
	1.05–1.13		1.05–1.13				1.01–1.07		1.02–1.12	
**Atrial fibrillation**	–	0.112	–	0.184	–	0.261	1.8	***0.024***	2.77	***0.012***
							1.08–2.99		1.25–6.16	
**LVOT obstruction** &	–	0.218	–	0.376	–	0.064	–	0.413	–	0.877
**NYHA functional class** #	–	0.975	–	0.765	–	0.289	2.09	0.001	–	0.367
							1.36–3.21			

SCD = sudden cardiac death, Max LV = maximum left ventricle, LVOT = left ventricular outflow tract, NYHA = New York Heart Association, HF = heart failure.

*Adjusted Multivariate Cox Proportional Hazards Analysis. Hazard risk based on multivariate Cox regression analysis including age, syncope, sudden death family history, maximum left ventricular wall thickness, left atrial diameter, atrial fibrillation, left ventricular outflow obstruction defined as gradient ≥30 mmHg at rest and NYHA functional class at initial evaluation.

# NYHA functional class II compared to class I.

& Patients with LVOT obstruction, who had not received septal reduction therapy (including myectomy and alcohol ablation) compared to all others; patients with syncope who had not received invasive treatment (including implantation of implantable cardioverter defibrillator, pace maker and septal reduction therapy) compared to all others.

**Figure 2:**
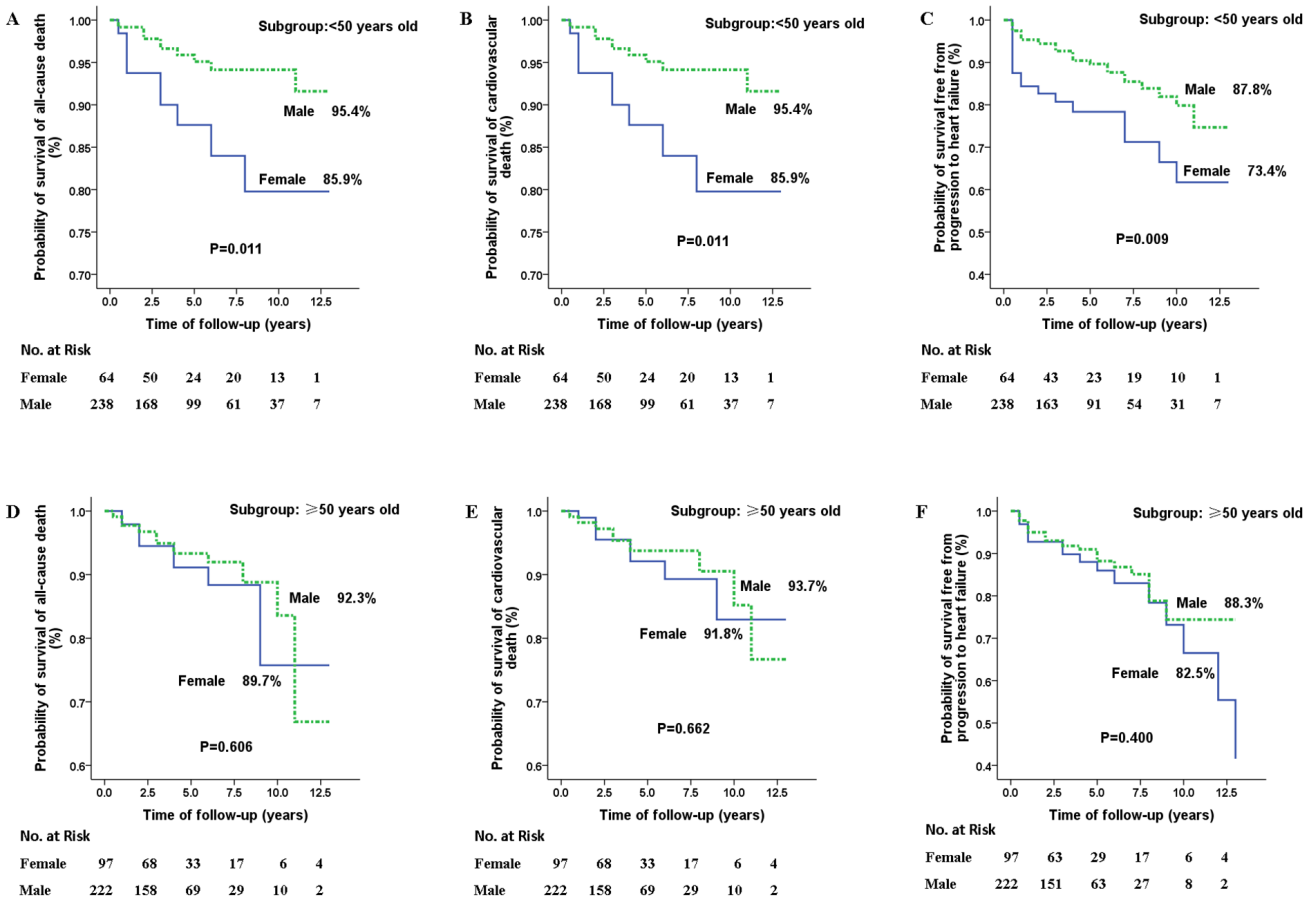
Survival in males and females free from death from all causes (A & B), cardiovascular death (C & D) and progression to heart failure (E & F), stratified by age (younger than 50 or 50 years and older). In patients <50 years old, female patients had greater rates of death from all causes (A), cardiovascular death (C) and progression to heart failure (E) than male patients. In patients ≥50 years old, no differences were found in the rates of death from all causes (B), cardiovascular death (D) and progression to heart failure (F). The P values were calculated by log-rank test.

To determine whether the observed differences between female and male patients were related to menopausal status, sub-analyses were performed in patients younger than 50 and those 50 and older. The elevated risk among female patients for death from all causes, cardiovascular death and progression to heart failure was identified only in patients <50 years old (*P = *0.011, 0.011 and 0.009, respectively) (Figure 2A,C,E), but not in patients ≥50 years old (*P = *0.606, 0.662 and 0.400, respectively) (Figure 2B,D,F). Among patients <50 years of age, females exhibited greater risks of all-cause mortality (HR 2.95 95% CI 1.22–7.13, *P = *0.016), cardiovascular death (HR 2.95 95% CI 1.22–7.13, *P = *0.016) and progression to heart failure (HR 2.15, 95% CI 1.18–3.92, *P = *0.012), compared to males.

**Figure 3:**
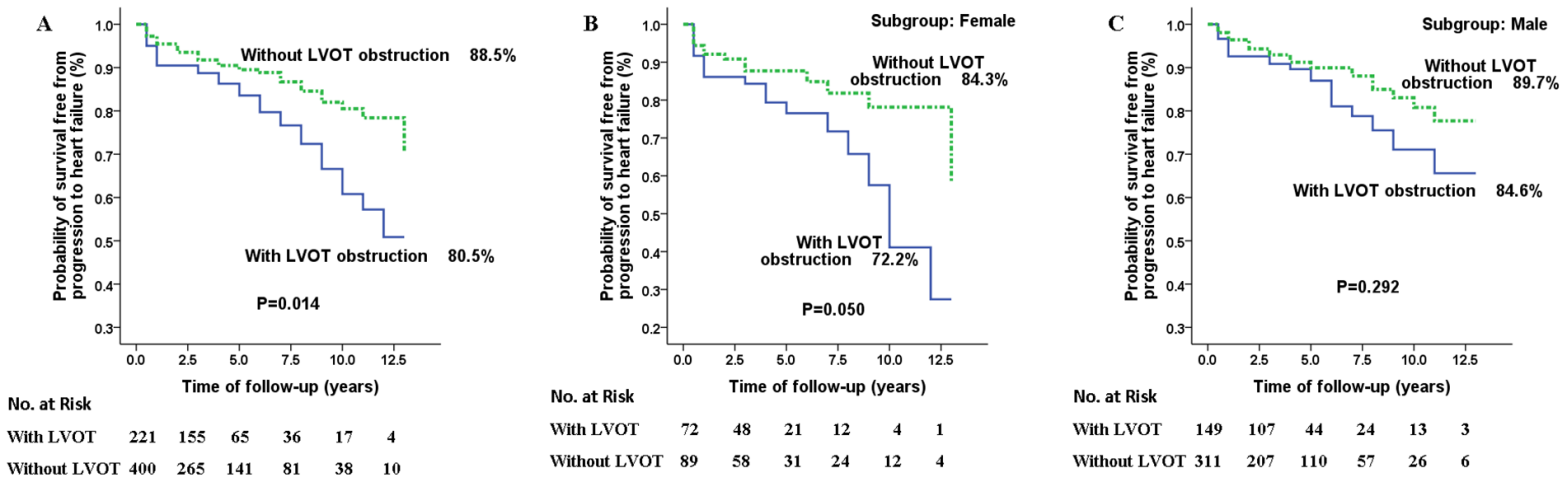
Survival in all patients (A), female patients (B), and male patients (C) with or without left ventricular outflow tract (LVOT) obstruction free from progression to heart failure. Patients with LVOT obstruction (excluding patients who had received septal reduction therapy) had a higher risk of progression to heart failure than patients without LVOT obstruction (A), but this difference was observed only in female patients (B). The P values were calculated by log-rank test.

Patients with LVOT obstruction (excluding patients who had undergone septal reduction therapy) were at higher risk for progression to heart failure than those without LVOT obstruction (*P = *0.014) (Figure 3A). Stratified by sex, the association of LVOT obstruction with progression to heart failure persisted only in female patients (*P = *0.050) (Figure 3B), and not male patients (*P = *0.292) (Figure 3C).

In multivariate Cox proportional hazard regression models, the left atrial diameter was a risk factor for death from all causes, cardiovascular death, progression to heart failure and stroke; AF was positively associated with progression to heart failure and stroke (Table 3).

The basic demographics, including average age, of the <50 years old subgroup and multivariate analysis results are similar to the whole cohort analysis results and shown in supplement (Table S1–3 in File S1).

## Discussion

This study investigated the association of sex with outcomes in HCM patients during a four year follow-up period. Female patients suffered higher risks of death from all causes, cardiovascular death and progression to heart failure, although significant differences between the sexes were observed only in patients younger than 50 years old. Heart function was more vulnerable to LVOT obstruction in female patients than in males.

In this study of 621 unrelated HCM patients evaluated at a single center, males comprised almost three quarters of the cohort. This distribution is consistent with most previous reports where male subjects constituted 55%–78% of HCM patients. [2], [3], [14]–[16] An equal proportion of males and females would be expected to manifest a highly penetrant autosomal dominant disease. Furthermore, while female patients in our cohort had more severe clinical manifestations, such as higher frequency and pressure gradient of LOVT obstruction, they were diagnosed approximately 3 years later than males. These discrepancies may be related to reduced awareness regarding cardiovascular risk among female patients, [17] fewer indications for medical screening programs and clinician bias, [18] or reluctance to seek medical attention until symptoms become more severe.

Because heart failure negatively correlates with survival for many kinds of cardiovascular disease, [19] we excluded patients with chronic heart failure at enrollment. During the follow-up period, female patients suffered more death from all causes, cardiovascular death and heart failure than did male patients. A previous study in a western population revealed a higher risk of compound events (including heart failure, death from heart failure and stroke) for women than men, but no difference in survival. [10] In that study, 10% of patients had chronic heart failure at enrollment, and the strong effect of severe heart failure (NYHA III/IV) on survival (HR 2.48, 95% CI 1.34–4.60, p = 0.004) may have masked the effect of sex. [10] While small sample study reported that female sex was an independent predictor of mortality in 163 Taiwanese with HCM, the proportion of heart failure at enrollment was not mentioned. [20] Our focus on patients at an earlier stage of HCM revealed the prognostic significance of sex on survival.

In our cohort, the higher rate of progression to heart failure in females was found to be related to the observed higher frequency of LVOT obstruction and increased LVOT gradient in females after a stratified analysis by sex, which is consistent with the finding in a previous study that female patients were more vulnerable than males to left ventricular remodeling due to LVOT obstruction. [21].

Sex differences in cardiovascular disease prognosis are thought to be related to differences in sex hormone signaling [22] and the expression of genes located on the sex chromosomes. [23]In animal and human studies of many cardiovascular disorders, [24], [25] females exhibited lower mortality and better outcomes. Estrogen appears to be protective in myocardial remodeling in response to volume overload, [24] pressure overload, [25] aging and acute myocardial ischemia, which results in better preservation of systolic cardiac function. In contrast, women appear to be more vulnerable to diastolic cardiac dysfunction than men. [26] Since diastolic dysfunction represents the earliest manifestation of HCM, [27]the propensity for diastolic dysfunction in women may explain their poorer outcomes. While hypertension is considered to be one reason for diastolic dysfunction, there was no significant difference in blood pressure between the females and the males in our cohort. We also adjusted for age when performing the multivariate analysis because older age is known to be a factor that can affect diastolic function. Recently, a mouse model of HCM demonstrated that estrogen was not always cardioprotective. [28] To determine the effect of estrogen, we performed sub-stratification by age. The significant differences in prognosis between the two sexes were evident only in patients <50 years old, implying that hormonal differences in premenopausal women could be responsible for this effect.

In addition, our study documented the predictive power of left atrial diameter on death from all causes, cardiovascular death, progression to heart failure and stroke. [12], [29] Each millimeter enlargement of the left atrium elevated the risk of adverse outcomes by 4–9%. Interestingly, although left atrial diameter is highly associated with AF, [30] we observed that its effect was independent from AF for the progression to heart failure and stroke. This finding implies that left atrial diameter may be used as a marker to predict the risk of heart failure and stroke accurately and stably because the types (paroxysmal, persistent and permanent), frequency and duration of AF are so variable and complicated.

Potential limitations of this study include the recruitment of subjects from a single center. However, Fuwai Hospital is the largest and most advanced cardiovascular referral hospital in China, so the subjects were derived from a large geographic area. Some patients preferred medication therapy to invasive therapy (including reduction therapy and ICD implantation) because of misgivings about potential complications and cost in China. Even for reduction therapy, alcohol septal ablation is always given priority over surgical myectomy for patients with LVOT obstruction in China. In our study, the higher proportion of events might be associated with the lower adoption of invasive therapy. On the other hand, the results obtained from this kind of HCM population that lacked invasive treatments should accurately reflect the natural disease course and be more informative.

In conclusion, our data show that female sex is associated with worse survival and heart failure in Chinese HCM patients. A high index of suspicion should be maintained in evaluating female patients for HCM. Further studies will be required to determine the mechanistic basis for these sex differences.

## Supporting Information

File S1 **Supporting tables.**
(DOC)
