# Supplementary material for: Female Sex Is Associated with Worse Prognosis in Patients with Hypertrophic Cardiomyopathy in China
Source: PLoS One. 2014 Jul 21;9(7):e102969. doi: 10.1371/journal.pone.0102969 (PMC4105411; doi:10.1371/journal.pone.0102969)
Supplement: File S1 — Supporting tables. (DOC) [file pone.0102969.s001.doc]

**Supplement**

| **Table S1.** Baseline Clinical Features of Group <50 years old According to Sex^*^ | | | | |  |
| --- | --- | --- | --- | --- | --- |
| **Parameters** | **Overall** | **Male Patients** | **Female Patients** | ***P* Value** |  |
| **Number of patients** | 302 | 238 (78.8%) | 64 (21.2%) |  |  |
| **Age at diagnosis (yr)** | 37.6 ± 9.6 | 38.3 ± 9.2 | 35.1 ± 10.7 | ***0.015*** |  |
| **Syncope** | 90 (29.8%) | 74 (31.1%) | 16 (25.0%) | 0.344 |  |
| **Coronary artery disease** | 14 (5.0%) | 12 (5.4%) | 2(3.6%) | 0.596 |  |
| **HCM family history** | 97 (32.1%) | 73 (30.7%) | 24(37.5%) | 0.299 |  |
| **SCD family history** | 48 (15.9%) | 35 (14.7%) | 13(20.3%) | 0.276 |  |
| **Heart rate (bpm)** | 72.2 ± 11.0 | 72.3 ± 11.4 | 71.6 ± 9.5 | 0.663 |  |
| **Systolic blood pressure (mmHg)** | 115.7 ± 14.8 | 117.4 ± 14.5 | 109.6 ± 14.7 | ***＜0.001*** |  |
| **Diastolic blood pressure (mmHg)** | 73.6± 10.5 | 74.8 ± 10.7 | 69.2 ± 8.5 | ***＜0.001*** |  |
| **Pathological Q wave** | 68 (22.5%) | 48 (20.2%) | 20 (31.3%) | 0.06 |  |
| **T wave inversion/Giant T wave** | 202 (66.9%) | 166 (69.7%) | 36 (56.3%) | 0.042 |  |
| **Maximum LV wall thickness (mm)** | 21.9 ± 5.1 | 21.7 ± 5.2 | 22.8 ± 4.7 | 0.118 |  |
| **Left atrial diameter (mm)** | 39.9 ± 7.5 | 39.8 ± 7.5 | 40.1 ± 7.6 | 0.822 |  |
| **LVEDD (mm)** | 43.4 ± 5.9 | 44.2 ± 5.7 | 40.4 ± 5.8 | ***＜0.001*** |  |
| **EF (%)** | 67.9 ± 8.2 | 68.0 ± 8.0 | 67.7 ± 8.9 | 0.778 |  |
| **LVOT gradient ≥ 30 mmHg** | 131 (43.4%) | 99 (41.6%) | 32 (50.0%) | 0.229 |  |
| **NYHA II** | 126 (41.7%) | 97(40.8%) | 29 (45.3%) | 0.512 |  |
| **VT/VF** | 4 (1.3%) | 2 (0.8%) | 2 (3.1%) | 0.156 |  |
| **Atrial fibrillation** | 24 (7.9%) | 18 (7.6%) | 6 (9.4%) | 0.634 |  |
| **Stroke** | 2 (0.7%) | 2 (0.8%) | 0 (0.0%) | 0.462 |  |
| **Transient HF** | 2 (2.0%) | 1 (0.4%) | 1 (1.6%) | 0.317 |  |
| **Acute myocardial infarction** | 1 (0.3%) | 1(0.4%) | 0 (0.0%) | 0.603 |  |
| **Surgical septal myectomy** | 4 (1.3%) | 2(0.8%) | 2 (3.1%) | 0.156 |  |
| **Alcohol septal ablation** | 46 (15.2%) | 37 (15.5%) | 9 (14.1%) | 0.769 |  |
| **ICD implantation** | 3 (1.0%) | 2(0.8%) | 1 (1.6%) | 0.605 |  |
| **Appropriate ICD discharge** | 2 (0.7%) | 2(0.8%) | 0 (0.0%) | 0.462 |  |
| **Pacemaker implantation** | 15 (5.0%) | 13 (5.5%) | 2 (3.1%) | 0.445 |  |
| **Medication** |  |  |  |  |  |
| **β-blocker** | 180 (80.8%) | 144 (78.7%) | 36 (85.7%) | 0.305 |  |
| **Calcium channel blocker** | 80 (36.5%) | 69 (39.0%) | 11 (26.2%) | 0.122 |  |
| HCM, hypertrophic cardiomyopathy; SCD, sudden cardiac death; Max LV, maximum left ventricular; LVEDD, left ventricular end-diastolic diameter; LVMI, left ventricular mass index; EF, ejection fraction; LVOT, left ventricular outflow tract; NYHA, New York Heart Association; VT/VF, ventricular tachycardia/fibrillation; HF, heart failure; ICD, implantable cardioverter defibrillator | | | | | |
| ^*^Data with normal distribution are presented as mean ± SD (standard deviation), while data with skewed distribution are presented as median (25-75% percentile), non-continuous variables expressed as proportions. | | | | | |

| **Table S2.** Cardiovascular Events and Invasive Treatments During Follow-up in Group <50 years old According to Sex^*^ | | | | |  |
| --- | --- | --- | --- | --- | --- |
| **Events** | **Overall** | **Male Patients** | **Female Patients** | ***P* Value** |  |
| **Death from all causes** | 20/302 (6.6%) | 11/238 (4.6%) | 9/64 (14.1%) | ***0.007*** |  |
| **Cardiovascular death** | 20/302 (6.6%) | 11/238 (4.6%) | 9/64 (14.1%) | ***0.007*** |  |
| **Sudden death** | 11/302 (3.6%) | 7/238 (2.9%) | 4/64 (6.3%) | 0.21 |  |
| **HF-related death** | 6/302 (2.0%) | 3/238 (1.3%) | 3/64 (4.7%) | 0.081 |  |
| **Fatal stroke** | 3/302 (1.0%) | 1/238 (0.4%) | 2/64 (3.1%) | 0.053 |  |
| **NYHA III/IV** | 46/302 (15.2%) | 29/238 (12.2%) | 17/64 (26.6%) | ***0.004*** |  |
| **VT/VF** | 3/298 (1.0%) | 2/236 (0.8%) | 1/62 (1.6%) | 0.591 |  |
| **Appropriate ICD discharge** | 0/300 (0.0%) | 0/236 (0.0%) | 0/64 (0.0%) | --- |  |
| **Atrial fibrillation** | 19/278 (6.8%) | 14/220(6.4%) | 5/58 (8.6%) | 0.545 |  |
| **Stroke** | 9/300 (3.0%) | 5/236 (2.1%) | 4/64 (6.3%) | 0.086 |  |
| **Transient HF** | 10/300 (3.3%) | 8/237 (3.4%) | 2/63 (3.2%) | 0.937 |  |
| **Acute myocardial infarction** | 0/301 (0.0%) | 0/237 (0.0%) | 0/64 (0.0%) | --- |  |
| **Surgical septal myectomy** | 9/298 (3.0%) | 8/236 (3.4%) | 1/62 (1.6%) | 0.467 |  |
| **Alcohol septal ablation** | 22/256 (8.6%) | 20/201 (10.0%) | 2/55 (3.6%) | 0.139 |  |
| **ICD implantation** | 2/299 (0.7%) | 1/236 (0.4%) | 1/63 (1.6%) | 0.314 |  |
| **Pacemaker implantation** | 21/287 (7.3%) | 17/225 (7.6%) | 4/62 (6.5%) | 0.768 |  |
| HF, heart failure; VT/VF, ventricular tachycardia/fibrillation; ICD, implantable cardioverter defibrillator; NYHA, New York Heart Association | | | | | |
| ^*^Proportion of patients for whom the event happened or who received treatment during follow-up (excluding patients with the specific event or who received the specific treatment at initial evaluation) | | | | | |

| **Table S3.** Relationship Between Clinical Variables at Initial Evaluation and Outcomes^*^ in Group <50 years old | | | | | | | | | | |
| --- | --- | --- | --- | --- | --- | --- | --- | --- | --- | --- |
|  | **Overall death** | | **Cardiovascular death** | | **SCD** | | **Chronic HF** | | **Stroke** | |
|  | Relative risk | *P* Value | Relative risk | *P* Value | Relative risk | *P* Value | Relative risk | *P* Value | Relative risk | *P* Value |
| **Variables** | (95% CI) |  | (95% CI) |  | (95% CI) |  | (95% CI) |  | (95% CI) |  |
| **Female sex** | 3.08 | ***0.026*** | 3.08 | ***0.026*** | --- | 0.659 | 2.09 | ***0.023*** | 8.62 | ***0.015*** |
|  | 1.14-8.282 |  | 1.14-8.282 |  |  |  | 1.11-3.93 |  | 1.52-49.09 |  |
| **Age at enrollment** | --- | 0.117 | --- | 0.117 | --- | 0.059 | --- | 0.294 | 1.21 | ***0.031*** |
|  |  |  |  |  |  |  |  |  | 1.02-1.45 |  |
| **Syncope^&^** | --- | 0.765 | --- | 0.765 | --- | 0.352 | --- | 0.562 | --- | 0.569 |
|  |  |  |  |  |  |  |  |  |  |  |
| **SCD family history** | --- | 0.053 | --- | 0.053 | 4.89 | ***0.03*** | --- | 0.544 | --- | 0.21 |
|  |  |  |  |  | 1.16-20.51 |  |  |  |  |  |
| **Maximum LV wall thickness** | --- | 0.494 | --- | 0.494 | --- | 0.614 | --- | 0.553 | --- | 0.34 |
|  |  |  |  |  |  |  |  |  |  |  |
| **Left atrial diameter** | 1.16 | ***＜0.001*** | 1.16 | ***＜0.001*** | 1.13 | ***0.002*** | --- | 0.693 | 1.21 | ***0.004*** |
|  | 1.10-1.22 |  | 1.10-1.22 |  | 1.05-1.22 |  |  |  | 1.06-1.37 |  |
| **Atrial fibrillation** | 0.15 | ***0.023*** | 0.15 | ***0.023*** | --- | 0.142 | 2.89 | ***0.002*** | 2.77 | 0.706 |
|  | 0.03-0.77 |  | 0.03-0.77 |  |  |  | 1.46-5.72 |  | 1.25-6.16 |  |
| **LVOT obstruction^&^** | --- | 0.372 | --- | 0.372 | --- | 0.826 | 1.93 | ***0.048*** | --- | 1.00 |
|  |  |  |  |  |  |  | 1.01-3.69 |  |  |  |
| **NYHA functional class^#^** | --- | 0.261 | --- | 0.261 | 9.6 | ***0.045*** | 2.65 | ***0.003*** | --- | 0.614 |
|  |  |  |  |  | 1.057-87.31 |  | 1.41-4.98 |  |  |  |
| SCD, sudden cardiac death; Max LV, maximum left ventricle; LVOT, left ventricular outflow tract; NYHA, New York Heart Association; HF, heart failure | | | | | | | | | | |
| ^*^Adjusted Multivariate Cox Proportional Hazards Analysis. Hazard risk based on multivariate Cox regression analysis including age, syncope, sudden death family history, maximum left ventricular wall thickness, left atrial diameter, atrial fibrillation, left ventricular outflow obstruction defined as gradient ≥ 30 mmHg at rest and NYHA functional class at initial evaluation. | | | | | | | | | | |
| ^#^NYHA functional class II compared to class I. | | | | | | | | | | |
| ^&^ Patients with syncope who had not received invasive treatment (including implantation of implantable cardioverter defibrillator, pace maker and septal reduction therapy) compared to all others; patients with LVOT obstruction, who had not received septal reduction therapy (including myectomy and alcohol ablation) compared to all others. | | | | | | | | | | |
